# BJPsych Bulletin In Memoriam List April 2026

**DOI:** 10.1192/bjb.2026.10224

**Published:** 2026-04

Benians, Robin Christopher, London, UK, FRCPsych 1983

Bonn, John Anthony, London, UK, FRCPsych 1981

Bloye, Darran James, Sheffield, UK, MRCPsych 1998

Burke, Aggrey Washington, Kingston upon Thames, UK, FRCPsych 2000

Daly, Joan Marion, Blackrock, Ireland, FRCPsych 1997

Enoch, Morgan David, Cardiff, UK, FRCPsych 1972

Gittleson, Neville Leon, Sheffield, UK, FRCPsych 1972

Kapp, Elinor Margaret, Cardiff, UK, FRCPsych 1992

Motichand, Sandeep, Milton Keynes, UK, Affiliate 2018

Owen, Bruce Mark, Newcastle upon Tyne, UK, MRCPsych 2000

Skarsten, Anders Roy Hargrave, South Perth, Australia, FRCPsych 2019

Wilson, James Harold, Epsom, UK, FRCPsych 2010

